# A Treatise on the Physiological and Moral Management of Infancy

**Published:** 1841-07

**Authors:** 


ART. IX.—*A Treatise on the Physiological and Moral Management of Infancy.* By ANDREW COMBE, M.D. &c. Second Edition.—*Edinb.* 1841. 8vo, pp. 380.

THE very high character given by us of this work on the appearance of the first edition, scarcely a year since, (see Br. and For. Med. Rev., vol. X., p. 241 and 514,) has been triumphantly confirmed by the profession and the public, as is proved by the demand for a new impression after so short a period. The volume was originally so complete that little room was left for improvement, and we believe this edition is substantially the same as the former. We feel it a duty to recommend Dr. Combe's Treatise in the most earnest manner to all our readers, and through them to all fathers and mothers, as the best guide extant for the physical and moral management of infants.

